# Network Meta-Analysis of Metabolic Effects of Olive-Oil in Humans Shows the Importance of Olive Oil Consumption With Moderate Polyphenol Levels as Part of the Mediterranean Diet

**DOI:** 10.3389/fnut.2019.00006

**Published:** 2019-02-12

**Authors:** Evangelia Tsartsou, Nikolaos Proutsos, Elias Castanas, Marilena Kampa

**Affiliations:** ^1^Laboratory of Experimental Endocrinology, School of Medicine, University of Crete, Heraklion, Greece; ^2^Hellenic Agricultural Organization Demeter, Athens, Greece

**Keywords:** olive oil, polyphenols, metabolism, Mediterranean diet, HDL, cholesterol, glucose

## Abstract

The beneficial role of olive oil consumption is nowadays widely recognized. However, it is not clear whether its health effects are due to the presence of monounsaturated lipids and/or to the antioxidant fraction of microconstituents present in olive oil. The aim of the present study was to analyze the exact role of olive oil in the modification of metabolic factors (glucose and circulating lipids) and explore the role of its antioxidant polyphenols. In the present work, we have performed a network meta-analysis of 30 human intervention studies, considering direct and indirect interactions and impact of each constituent. Interestingly, we show that the impact of olive oil on glucose, triglycerides, and LDL-cholesterol is mediated through an adherence to the Mediterranean diet, with the only notable effect of olive oil polyphenols being the increase of HDL-cholesterol, and the amelioration of the antioxidant and inflammatory status of the subjects. Additionally, we report for the first time that lower antioxidant polyphenol levels may be sufficient for the beneficial effects of olive oil, while we show that the lipid fraction of olive oil may be responsible for some of its beneficial actions. In all parameters examined the beneficial effect of olive oil was more pronounced in subjects with an established metabolic syndrome or other chronic conditions/diseases. In conclusion, all these findings provide new knowledge that could lead to re-establishment of the role of olive oil in human nutrition.

## Introduction

Impaired glucose and lipid metabolism together with increased blood pressure, that characterize a pro-inflammatory state (1–3) lead to an increased likelihood of insulin resistance/type 2 diabetes, and atherosclerosis/cardiovascular disease (2), which are the commonest metabolic dysfunctions in humans. These alterations, together with a resulting pre-thrombotic state (3) may result in premature death. Since 1979, after the milestone publication of Keys et al. (4), it was evidenced that the Mediterranean-type of diet resulted in a reduced risk for cardiovascular disease, despite its high (especially mono-unsaturated) lipid content, of which olive oil is the main source. Indeed, a large body of scientific evidence confirmed the benefits of Mediterranean diet and olive oil consumption, on the lipid profile, lipid and DNA oxidation, insulin resistance, and inflammation (5–8), resulting in a decreased cardiovascular risk.

Olive oil is a functional food that, besides its high content in mono-unsaturated fatty acids, also contains other minor, biologically active, components, such as vitamins, minerals, and polyphenols (9). The quality of olive oil is mainly defined by EU regulations (2568/91/EEC & 1019/2002/EC), which state the requirements for each commercial type: extra-virgin olive oil (produced by direct-press or simple, low-speed, centrifugation methods), virgin, common or refined olive oil. However, even extra-virgin olive oil may differ in terms of its microconstituents, related to the method of isolation, micro-climate, and cultivation conditions.

Health effects of olive oil, primarily as a constituent of the Mediterranean diet, has been the subject of many studies (10–22) and were mainly attributed to its polyphenol content. Antioxidant and anti-inflammatory properties and improvement in endothelial dysfunction and lipid profile have been reported for dietary polyphenols (21, 23).

Here, we have used published human intervention studies using olive oil, totalizing 7688 subjects, mostly out of the context of the Mediterranean diet and performed a network meta-analysis of metabolic changes in circulating glucose and lipid parameters, in relation to the polyphenol load of the oil. Network meta-analysis reports a final effect of a given intervention, considering both direct and indirect interactions. In addition, we extracted data related to inflammation and oxidation status and analyzed them separately, in a narrative review, as the number of studies was small. We provide novel data on the effect of olive oil and suggest a possible paradigm shift for its use in human nutrition.

## Materials and Methods

**Figure 1:**
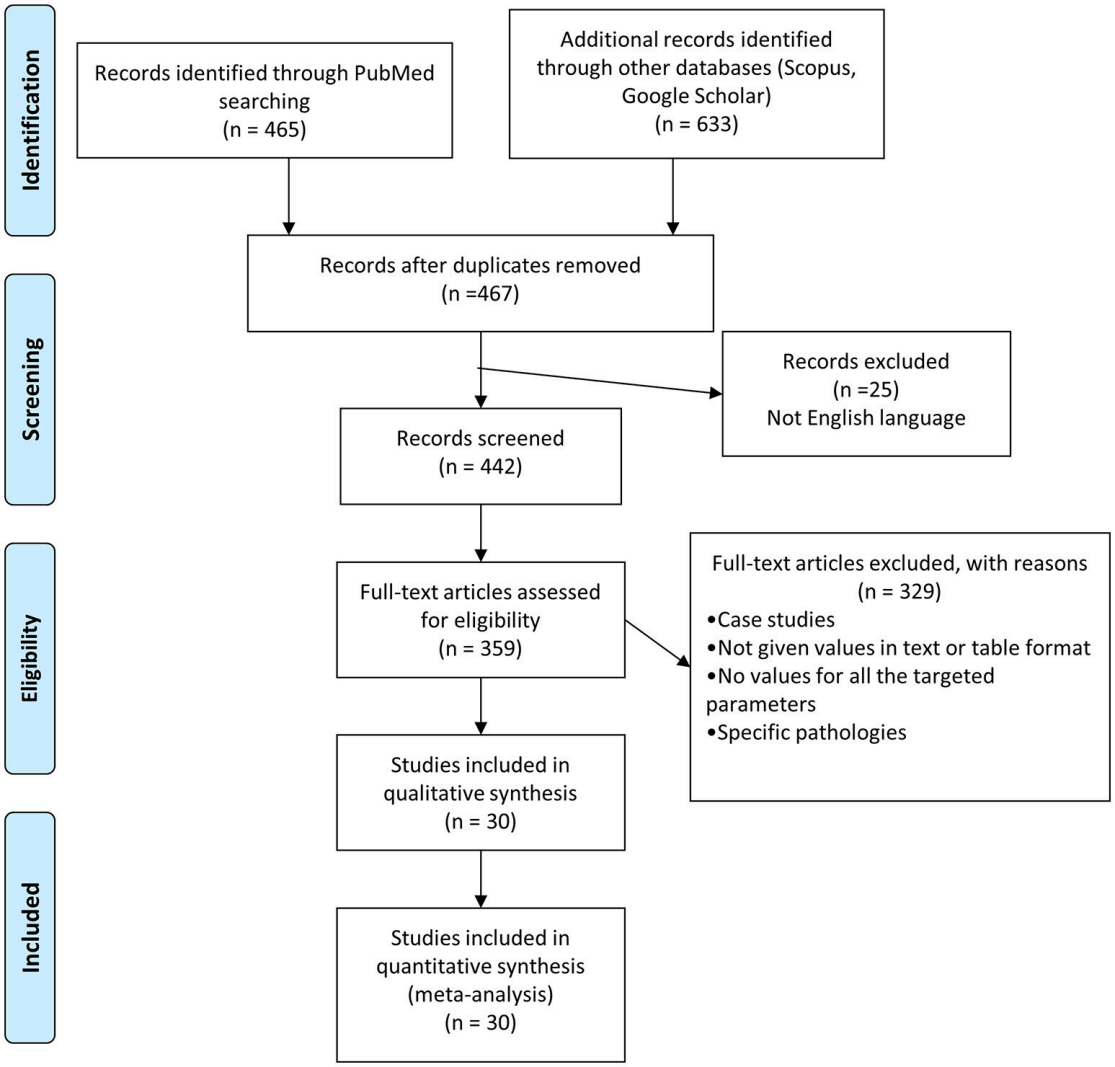
PRISMA flow chart of the search strategy used in this study.

Studies were retrieved from PubMed, Scopus and Google Scholar, using the terms: “(olive oil) AND (intervention study) AND diet AND human” at 20 September 2017. A total of 465 unique records were retrieved (excluding reviews) and scanned for changes of metabolic (glucose, total-, HDL- and LDL cholesterol, triglycerides), markers. The search strategy and details of the excluded studies are presented in Figure 1 (PRISMA flow chart). Details of the retained studies are presented in Table 1. Two or more categories were extracted from each study: (1) Normal (control) diet (CD) or Control Sample (CS); (2) Intervention including Mediterranean Diet with olive oil containing low or high polyphenols; (3) Intervention including olive oil with a low (<60 mg/kg) polyphenol content (LPC); (4) Diet including different categories of olive oil (for example extra-virgin vs. virgin or refined olive oil, etc.). Since our study is a meta-analysis and systematic review of human intervention studies it does not necessitates an ethical committee approval.

**Table 1:** Description of the studies used in the present analysis.

**No**	**Number of participants**	**Age range**	**Male: female ratio (%)**	**Duration**	Types of oil/diet^*^	**Intervention groups (IGs)-treatments (TG)**	**References**
1	90	20–50	28.9:71.1	3 months	2 + 5	•CD (habitual diet) •TMD + VOO with PC 328 mg/kg •TMD + WOO with PC 55 mg/kg	(24)
2	200	33.1 ± 10.6	100:0	13 weeks	2 + 3	•25 ml/d OO with PC 366 mg/kg •25 ml/d OO with PC 164 mg/kg •25 ml/d OO with PC 2.7 mg/kg	(25)
3	28	68 (6.45)	100:0	10 weeks	2 + 3	•50 ml/d OO with PC 161 mg/kg •50 ml/d OO with PC 14.67 mg/kg	(26)
4	180	44.3 (6.4)	55:45	24 months	5	•CD •MD	(27)
5	3,042	18–89	49.77:50.23	20 months	5	•MD	(28)
6	30	61 ± 19.2	100:0	15 weeks	2 + 3	•25 ml/d OO with PC 150 mg/kg •25 ml/d OO with PC 68 mg/kg •25 ml/d OO with PC 0 mg/kg	(29)
7	10	23–30	100:0	2 weeks	2	•50 g/d OO supplementation	(30)
8	200	20–60	100:0	15 weeks	2	•25 ml/d OO with PC 366 mg/kg •25 ml/d OO with PC 164 mg/kg •25 ml/d OO with PC 2.7 mg/kg	(9)
9	22	18–65	54.55:45.45	22 weeks	1 + 3	•40 ml/d OO with PC 166 mg/l •40 ml/d OO with PC 2 mg/l	(31)
10	46	18–58	36.96:69.57	16 weeks	1	•69 g/d OO with PC 308 mg/kg •69 g/d OO with PC 43 mg/kg	(32)
11	24	69.9 ± 2.1	100:0	9 months	1 + 3	•40–42 g/d OO with PC 800 mg/kg •40–42 g/d OO with PC 60 mg/kg	(33)
12	10	42–67	80:20	10 weeks	1 + 3	•20 g/d OO with PC 238 mg/kg •20 g/d OO with PC 11 mg/kg	(34)
13	6	27–33	100:0	~2 months	3	•50 ml/d OO with PC 487.5 mg/L •50 ml/d OO with PC 975 mg/L •50 ml/d OO with PC 1462.5 mg/L •50 ml/d OO with PC 1950 mg/L	(35)
14	12	25 ± 3	100:0	~68 days	1 + 3 + CO	•50 ml/d OO with PC 607 ppm •50 ml/d OO with PC 16 ppm •50 ml CO	(36)
15	12	20–22	100:0	~6 weeks	1 + 3	•25 ml/d OO with PC 486 mg/kg •25 ml/d OO with PC 133 mg/kg •25 ml/d OO with PC 10 mg/kg	(37)
16	182	20–60	100:0	15 weeks	2 + 3	•25 ml/d OO with PC 366 mg/kg •25 ml/d OO with PC 164 mg/kg •25 ml/d OO with PC 2.7 mg/kg	(38)
17	25	30 ± 9,1%	44–56	10 weeks	1	•70 g/d OO with PC 308 mg/kg •70 g/d OO with PC43 mg/kg	(39)
18	18	22–61	50:50	18 weeks	6	•Standard Diet •OED + 1,200 mg/d a tocopherol •LED + 1,200 mg/d a tocopherol	(40)
19	21	59 (53–68)	23.8:76.2	~10 weeks	2	•40 ml/d OO with PC 400 ppm •40 ml/d OO with PC 80 ppm	(41)
20	10	46–67	0:100	32 weeks	1	•50 g/d OO with PC 592 mg/kg •50 g/d OO with PC 147 mg/kg	(42)
21	28	19–31	100:0	77 days	3 + 6	•80 g/d CO •68 g /d OO + 12 g/d SO	(43)
22	32	29.6 ± 10.3	100:0	9 weeks	3 + 6	•OO •palm olein •lard	(44)
23	47	33.5 ± 10.9	100:0	10 weeks	3	•25 ml/d OO with PC 366 mg/kg •25 ml/d OO with PC 2.7 mg/kg	(45)
24	18	56 ± 5	0:100	56 days	1 + 6	•SFD (50 g/d butter) •MFD (50cc /d EVOO).	(46)
25	102	51.45 ± 8.27	20.59:79.41	~90 days	1 + 6	•Control (usual diet) •3 g/d FO •10 mL/d OO •10 ml/d OO + 3 g/dFO	(47)
26	33	35–80	57.57:42.43	15 weeks	1 + 2	•25 ml/d VOO with PC 80 ppm •25 ml/d FVOO with PC 500 ppm •25 ml/d FVOOT with PC 500 ppm	(48)
27	25	20–59	100:0	10 weeks	2 + 3	•25 ml/d OO with PC 366 mg/kg •25 ml/d OO with PC 2.7 mg/kg	(49)
28	3,042	18–89	49.77:50.23	10 years	5	•MD (Evaluation Model-review study)	(50)
29	33	35–80	57.58:42.42	~15 weeks	2	•25 ml/d VOO with PC 80 ppm •25 ml/d FVOO with PC 500 ppm •25 ml/d FVOOT with PC 500 ppm	(51)
30	160	33.3 ± 11.1	100:0	13 weeks	3	•25 ml/d OO with PC 366 mg/kg •25 ml/d OO with PC 164 mg/kg •25 ml/d OO with PC 2.7 mg/kg	(52)

* Intervention with OO, as defined by EU Regulation (2568/91/EE): 1. EVOO, 2. VOO, 3. Common OO or ROO, 4. OPO, 5. MD /TMD, 6. Other Oils/Diets.

*OO, Olive Oil; PC, Polyphenol Content; EVOO, Extra Virgin Olive Oil; VOO, Virgin Olive Oil; OPO, Olive Pomace Oil, ROO, Refined Olive Oil; CD, Control Diet; TMD, Traditional Mediterranean Diet, FVOO. Functional Virgin Olive Oil; FVOOT, Functional Virgin Olive Oil with 50% phenolic content from Thyme; SFD, Saturated fat diet; MFD, Monounsaturated Fat Diet; cOO, complementary Olive Oil; FO, Fish Oil; CO, Corn Oil; WOO, Washed virgin Olive Oil*.

From each study, the mean, standard deviation and number of participants per group were extracted and tabulated in Excel. Meta-analysis was performed in Excel, using the free MetaXL V5.3 (www.epigear.com) add-on. Network Meta-Analysis (or indirect method meta-analysis) (53), was performed, using fixed effects. In a network meta-analysis, the effect of two treatments is measured, that each was compared against a similar control group in a meta-analysis. For example, if treatment A and treatment B were directly compared vs. placebo in separate meta-analyses, we can use these two pooled results to get an estimate of the effects of A vs. B in an indirect comparison as effect A vs. Placebo minus effect B vs. Placebo. Here, we report both direct and indirect interactions of each intervention (see Supplemental Tables 1–6) and the combined effect was calculated and reported. In addition, results of individual studies per treatment were also calculated. The inverse variance was always used in both analyses and results are presented as a standardized mean difference per study, sub-group and network, expressed as Cohen's standardized d (the difference between the means divided by the standard deviation for the data), with 95% confidence interval (95% CI).

In the 30 retained studies, a further search for an oxidative and inflammatory marker was performed. The authors of the retrieved documents use a plethora of parameters to access lipid oxidation and changes in other oxidation and inflammatory parameters. In addition to oxidized LDL (oxLDL) (assessed in 7 studies and analyzed through meta-analysis), all other inflammation and oxidation parameters were reported in a small number of studies. In view of their small number, a meta-analysis was not possible for these markers and therefore a comparison of percent differences was performed with the SPSS V21 program. A significance of 0.05 was retained as a significance threshold.

## Results

### Description of the Studies

Most of the included studies were randomized cross-over controlled clinical trials with dietary interventions (Table 1). The characteristics of participants were: healthy subjects in the majority (70%) of the studies. Participants with an established metabolic syndrome were reported in 2 studies and with hypercholesterolemia in 6 studies, while patients with an established cardiovascular or peripheral vascular disease were reported in one study. Postmenopausal women, which were reported in 2 studies, were included in the healthy participant's category. Only males participated in 15 studies and only women in 2 studies, while both sexes were reported in 13 studies. Eleven studies covered a wide range of ages from 18 to 89 years, others (10) focused only on young adults (aged 18–40 years), others (7) examined middle-aged (45–60 years) and the rest of the studies (2) focused on old aged participants >65 years. In most studies (21), the number of participants ranged from 10 to 100 (with exception one study that it was <10), in others (4) the number of participants ranged from 100 to 200 and it was >200 in 4 studies.

The intervention period of most studies (40%), including the washout periods, lasted 2–3 months (30%), 3–12 months (20%), and in 10% the intervention period was over 1 year. The dietary interventions included the consumption of olive oil with a different polyphenol content. In most studies (57%), participants consumed extra- or virgin olive oil high in polyphenols (HPOO), over common or refined olive oil, low in polyphenols (LPOO). In addition, several studies (20%) used extra- or virgin olive oil, in comparison with other oils (corn, fish, etc.).

In most studies (36.6%) participants received a daily dose of 25 ml olive oil and 15 ml in one study. In some studies (16.6%), the participants received a daily olive oil dose of 40 ml (2 studies) and 50 ml (3 studies) and in the rest of the studies (10%), the participants received 68–70 g of olive oil per day.

### Effect of Olive Oil on Metabolic Parameters

**Figure 2:**
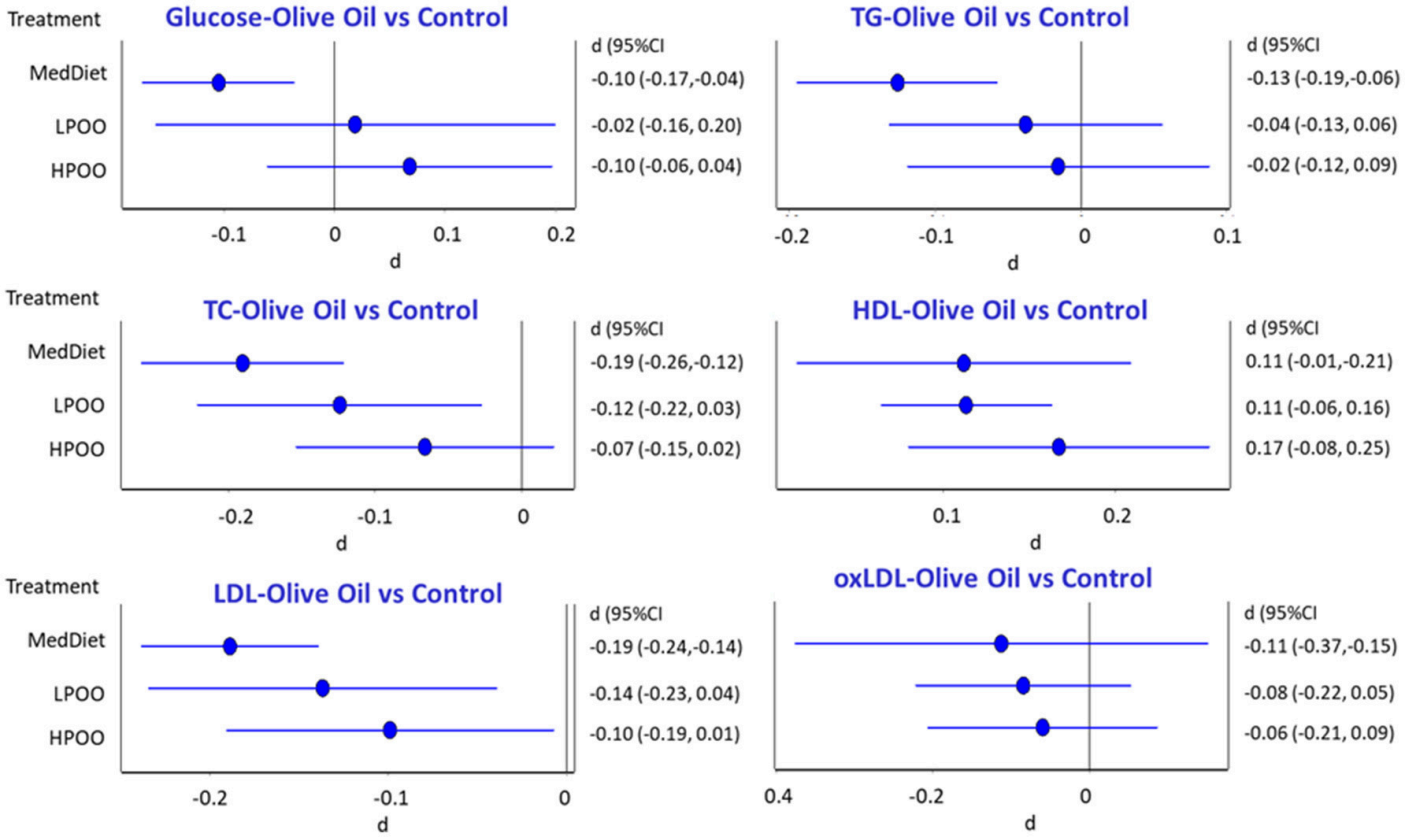
Network meta-analysis of circulating glucose (Glu), triglyceride (TC), total (TC), HDL- (HDL), LDL-cholesterol (LDL), and oxidized LDL (oxLDL) circulating levels in the 30 human studies, presented in Table 1. HPOO stands for olive oil high in polyphenols, while LPOO presents data of olive oil, low in polyphenols. Standardized differences from a control (westernized) diet are presented, together with their 95% confidence intervals.

The network meta-analysis revealed that adherence to the Mediterranean diet significantly decreased circulating glucose levels (Standardized difference *d* = −0.105, 95%CI = −0.174, −0.036), total cholesterol (*d* = −0.191, 95%CI = −0.259, −0.122), LDL-cholesterol (*d* = −0.189, 95%CI = −0.238, −0.140) and oxidized LDL levels (*d* = −0.112, 95%CI = −0.375, 0.150), as compared to the control, westernized diet. Intervention with olive oil decreased this change gradually, related to its polyphenol content. In addition, adherence to the Mediterranean diet significantly increased HDL-cholesterol (*d* = 0.113, 95%CI = 0.064, 0.163). However, low polyphenol content olive oil did not further modify HDL levels, while high polyphenol olive oil increased HDL-cholesterol concentration by almost 50% (*d* = 0.163, 95%CI = 0.080, 0.255). Figure 2 depicts these network meta-analysis changes, while Supplemental Figures 1–6 present the analysis of individual studies.

Analyzing further the direct and indirect contribution of the Mediterranean diet adherence and the effect of high and low-polyphenol content olive oil in these changes (see the Materials and Methods section for details of the used methodology and Supplemental Tables 1–6) it becomes apparent that the main driver for glucose, triglycerides, total and LDL cholesterol is the Mediterranean diet *per se*, suggesting that this impact on cardio-metabolic biochemical indices may be driven by a number of constituents, in addition to those included in the olive oil (see for example the direct effect of HPOO on LDL levels (*d* = −0.074, as compared to its indirect effect, when the Mediterranean diet is also included (*d* = −0.116), Supplemental Table 5). In contrast, the effect of olive oil on HDL concentration is directly related to its polyphenol content, as detailed in Supplemental Table 4.

In conclusion, a network meta-analysis of olive oil intervention studies in humans suggests that the only direct impact of olive oil polyphenols in cardio-metabolic biochemical indices is its direct effect of HDL-cholesterol levels, while other parameters (blood glucose, triglyceride, LDL) are modified because of the Mediterranean diet, in which olive oil is a major constituent. In addition, our data confirm the beneficial effect of the Mediterranean toward a westernized diet pattern.

### Effect of Olive Oil on Oxidative Parameters

An increased resistance of LDL to oxidation is reported in 5/6 studies, a decrease in LDL oxidation rate is found in 3/4 studies, and the detection of antibodies against oxLDL was also decreased by intervention with olive oil (Table 2). However, no correlation between polyphenol content and the resistance of LDL to oxidation was found. These data suggest that a moderate polyphenol content may be sufficient to protect LDL from oxidation. Further evidence for this is reflected by the decrease of hydroxyl fatty acids and the Lipoprotein lipase gene expression, which increased significantly (+26%) after a high polyphenol olive oil intervention (49), suggesting a direct effect of polyphenols to the transcription of lipid enzyme regulation. These data further verify our network meta-analysis of oxLDL, presented above, which indicates that the major impact on oxLDL is the adherence to the Mediterranean diet, further suggesting that many of the olive oil-attributed benefits might be related to other microconstituents of elements of this diet, in addition to olive oil polyphenols.

**Table 2:** Percentage (absolute) changes in the oxidative parameters related to the LDL oxidation after treatment with olive oil with different phenolic content (oxLDL is presented in the network meta-analysis section).

**Change**	**Treatment**	**Polyphenolic content**	**Daily dose**	**References**
**RESISTANCE OF LDL TO OXIDATION**				
***a. Lagtime (min)***				
+7% (+8)	CS vs. OO (HPC)	150 mg/kg	25 ml	(29)
+5.01%	CS vs. OO (HPC)	366 mg/kg	25 ml	(49)
+3.4% (+4)	CS vs. OO (LPC)	68 mg/kg	25 ml	(29)
+3.17%	CS vs. OO (LPC)	2.7 mg/kg		(49)
−1,45% (−1.6)	OO (HPC) vs. OO (LPC)	308 vs. 43 mg/kg	69 g	(32)
+14,8% (+13)	OO (HPC) vs. OO (LPC)	238 vs. 11 mg/kg	20 g	(34)
+0.7% (+0.83)	OO (HPC) vs. OO (LPC)	308 vs. 43 mg/kg	70 g	(39)
***b. Oxidation rate (μmol dienes min**^**−*1***^**g**^**−*1***^**of LDL or HDL protein)***				
−106% (−0.06 LDL protein)	CS vs. OO (HPC)	150 mg/kg	25 ml	(29)
no change	CS vs. OO (HPC)	366 mg/kg	25 ml	(49)
−0.9% (−0.05 LDL protein)	CS vs. OO(LPC)	68 mg/kg	25 ml	(29)
no change	CS vs. OO (LPC)	2.7 mg/kg		(49)
−0.8% (−0.1 max rate HDL protein)	OO (HPC) vs. OO (LPC)	308 vs. 43 mg/kg	69 g	(32)
no change (max rate)	OO (HPC) vs. OO (LPC)	308 vs. 43 mg/kg	70 g	(39)
***c. Antibodies against oxLDL (U/L)***				
−17% (−187)	CS vs. OO (HPC)	150 mg/kg	25 ml	(29)
−2.3% (−18)	CS vs. OO (LPC)	68 mg/kg	25 ml	(29)
**Hydroxy fatty acids** ***(nmol/L)***				
−3.3% (−41)	CS vs. OO (HPC)	366 mg/kg	25 ml	(9)
−2.6% (−31)	CS vs. OO (LPC)	2.7 mg/kg	25 ml	(9)
−10.6% (−19)	OO (HPC) vs. OO (LPC)	366 vs. 2,7 mg/kg	25 ml	(9)
**LPL gene expretion (%)**				
+26%	CS vs. OO (HPC)	366 mg/kg	25 ml	(49)
no change	CS vs. OO (LPC)	2.7 mg/kg		(49)

*CD, Control Diet; TMD, Traditional Mediterranean Diet; CS, Control Sample; OO, Olive Oil; LPC, Low Polyphenol Content; HPC, High Polyphenol Content; cOO, complementary Olive Oil*.

DNA oxidation parameters (oxidized DNA bases, DNA breaks and 8-oxo-dG in urine) were decreased by 3–61% after olive oil intervention (Table 3). Here too, no direct correlation with the polyphenol content was observed, suggesting that a moderate content of polyphenols (calculated by linear regression to 56 mg polyphenols/L olive oil) may be sufficient to prevent nucleic acid oxidation. Corroborating to this effect, the plasma antioxidant capacity (measured by different methods, accessing discrete antioxidant molecules in the plasma/serum) presented an increase after olive oil intervention, indicative of an enhanced role of polyphenols in increasing the organism's antioxidant capacities. Here too, the effect is also observed with low polyphenol content (necessary dose of 98 mg/L), suggesting that other elements may be also interfering with the observed decrease.

**Table 3:** Percentage (absolute) changes in the Parameters of the oxidative stress and oxidative damage of DNA& RNA after Treatment with Olive Oil with different phenolic content.

**Change**	**Treatment**	**Polyphenolic content**	**Daily dose**	**References**
**DNA** **+** **RNA OXIDATION**				
***a. Oxidized DNA bases(% DNA in comet tail)***				
−52.9% (−6.3)	CS vs. OO (HPC)	592 mg/kg	50 g	(42)
−45.4% (−5.4)	CS vs. OO (LPC)	147 mg/kg	50 g	(42)
***b. Basal DNA breaks (% DNA in comet tail)***				
−60.5% (−7.8)	CS vs. OO (HPC)	592 mg/kg	50 g	(42)
−61.2% (−7.9)	CS vs. OO (LPC)	147 mg/kg	50 g	(42)
**DNA OXIDATION BIOMARKER: 8-OXO-DG IN URINE**				
−13%	CS vs. OO (HCP)	366 mg/kg	25 ml	(38)
−12.8% (−1.48)	CD vsTMD (HPC)	328 mg/kg		(24)
−3.7% (−0.41)	CD vs. TMD (LPC)	55 mg/kg		(24)
−51.67%) (−8.56)	CS vs. OO (HPC)	486 mg/kg	25 ml	(37)
−30.8% (−3.58)	CS vs. OO (LPC)	10 mg/kg	25 ml	(37)
**PLASMA ANTIOXIDANT CAPACITY** ***(μM Cu**^**++**^**REDUCED)***				
~+40% (+81)	CS vs. OO (HPC)	166 mg/l	40 ml	(31)
+14.75% (+12.27)	CS vs. OO (HPC)	607 ppm	50 ml	(36)
No change	CS vs. OO (HPC)	592 mg/kg	50 g	(42)
−28% (−60)	CS vs. OO (LPC)	2 mg/L		(31)
+8,21% (+7,17)	CS vs. OO (LPC)	16 ppm	50 ml	(36)
−11.1% (−0.1 mmol/l)	CS vs. OO (LPC)	147 mg/kg	50 g	(42)
**FRAP*****(mmol/l)***				
+0.6% (+0.006)	OO (HPC) vs. OO (LPC)	308 vs. 43 mg/kg	69 g	(32)
No change	OO (HPC) vs. OO (LPC)	308 vs. 43 mg/kg	70 g	(39)

*CD, Control Diet; TMD, Traditional Mediterranean Diet; CS, Control Sample; OO, Olive Oil; LPC, Low Polyphenol Content; HPC, High Polyphenol Content*.

### Effect of Olive Oil on Inflammatory Markers

High sensitivity CRP decreased in all, but one studies after the olive oil-high polyphenol content intervention (Table 4). The biggest reductions (−39.4 and −35.86%) in CRP were observed after instauration of a Mediterranean diet profile with the inclusion of olive oil, regardless of its polyphenol content. It is also to note that in one of these investigations, the target group was patients with a diagnosed metabolic syndrome, while in another one, patients with cardiovascular disease were included. Finally, several cytokines were also decreased after olive oil ± Mediterranean diet intervention.

**Table 4:** Percentage (absolute) changes in the inflammatory parameters related to CVD risk factors after treatment with olive oil with different phenolic content.

**Change**	**Treatment**	**Polyphenolic content**	**Daily dose**	**References**
**IFN-γ*****(pg/ml)***				
−9.25% (−2.5)	CD vs. TMD (HPC)	328 mg/kg		(24)
No change	CD vs. TMD (LPC)	55 mg/kg		(24)
**CRP*****(mg/dl)***				
−28.6% (−0.02)	CD vs. TMD (HPC)	328 mg/kg		(24)
−39.3% (−0.11)	CS vs. MD (HPC)		8 g	(27)
+40.8% (+0.2)	CS vs. OO			(44)
+148.6% (+1.56)	CS vs. OO (HPC)		10 ml	(47)
−27.2% (−0.03)	CD vs. TMD (LPC)	55 mg/kg		(24)
−3.44% (−0.01)	CS vs. MD (LPC)			(27)
−39.4% (−0.063)	OO (HPC) vs. OO (LPC)	161 vs. 14.67 mg/kg	50 ml	(26)
−35.86% (−0.1)	CD (HPC) vs. MD (LPC)			(27)
−20% (−0.04)	MD (LTS) vs. MD (HTS)			(28)
+38.8% (+0.19)	CS vs. Palm olein			(44)
+8.16% (+0.04)	CS vs. Lard			(44)
**IL-6*****(pg/ml)***				
−33.3% (−0.7)	CS vs. MD (HPC)		8 g	(27)
−5.7% (−0.1)	CS vs. MD (LPC)			(27)
−12.03% (−0.166)	OO (HPC) vs. OO (LPC)	161 vs. 14.67 mg/kg	50 ml	(26)
−27.6% (−0.6)	CD (HPC) vs. MD (LPC)		8 g	(27)
−17% (−0.65)	MD (LTS) vs. MD (HTS)			(28)
**IL-7*****(pg/ml)***				
−20.8% (−0.5)	CS vs. MD (HPC)		8 g	(27)
No change	CS vs. MD (LPC)			(27)
−20.8% (−0.5)	CD (HPC) vs. MD (LPC)		8 g	(27)
**IL-18*****(pg/ml)***				
−11.4% (−19)	CS vs. MD (HPC)		8 g	(27)
−2.3% (−4)	CS vs. MD (LPC)			(27)
−9.10% (−15)	CD (HPC) vs. MD (LPC)		8 g	(27)
**TXB2 (Thromboxane B2)** ***(ng/ml Serum)***				
−21%	CS vs. OO (HPC)	166 mg/l	40 ml	(31)
−21.9% (−112.7)	CS vs. OO (HPC)	607 ppm	50 ml	(36)
+38.15% (+286.06)	CS vs. CO		50 ml	(36)
+21%	CS vs. OO (LPC)	2 mg/l	40 ml	(31)
+83.4% (+451.14)	CS vs. OO (LPC)	16 ppm	50 ml	(36)

*CD, Control Diet; TMD, Traditional Mediterranean Diet; CS, Control Sample; OO, Olive Oil; LPC, Low Polyphenol Content; HPC, High Polyphenol Content; cOO, complementary Olive Oil; FO, Fish Oil; CO, Corn Oil, LTS, Lowest Tertile Score; HTS, Highest Tertile Score*.

## Discussion

Diets following the principles of the Mediterranean diet, rich in vegetables, legumes, and olive oil have proven their beneficial character in preventing cardiovascular disease (54), diabetes (55–58), and hyperlipidemia (59). These effects were mainly attributed to the antioxidant fraction of microconstituents present in olive oil and in other elements of this diet (fruits, vegetables). In fact, a meta-analysis of 50 studies and 534,906 individuals revealed that adherence to the Mediterranean diet was associated with reduced risk of metabolic syndrome (60) and their participants expressed lower levels of inflammatory markers related to atherosclerosis (61).

The beneficial role of olive oil consumption is nowadays widely recognized, with the European Food Safety Authority **(**EFSA) approving two health claims regarding olive oil (62). They suggest its use to replace saturated fats to keep normal blood cholesterol levels and protect blood lipids from oxidative stress, with the later effect to be achieved by olive oil polyphenols contained in a daily intake of 20 g of extra-virgin olive oil.

In order to delineate the exact role of olive oil in the above metabolic changes, we have performed a network meta-analysis of 30 human intervention studies. Network meta-analysis evaluates, in addition to the direct effects of each treatment/intervention, the indirect effects resulting from the linear interaction between the network components. For example, the effect of HPOO on glucose levels, shown in the scheme below Supplemental Table 1 is the integrator of its direct effect (line control-HPOO) and the indirect effects (control-LPOO-HPOO and Control-MedDiet-HPOO), while the impact of the MedDiet in the same scheme is its direct effect on glucose (line Control-MedDiet) and the sum of its indirect effects (Control-HPOO-MedDiet and Control-LPOO-MedDiet) (see Salanti et al. (53) and references therein for a thorough description of the Network meta-analysis theory and applications). Using this approach, we show that the effect of olive oil on glucose and circulating lipids cannot be distinguished from the effect of adherence to a Mediterranean diet pattern, while the only clear-cut effect of a high-polyphenol olive oil is its effect on HDL-cholesterol. In this respect, we confirm our recent findings (63) that olive polyphenols, administered in a functional food and at a dose compatible with the EFSA-suggested dose of olive oil, do not modify the circulating glucose levels, while they ameliorate insulin sensitivity. These results were further corroborated by the reported direct protective effect of polyphenols in the pancreas (64) and the amelioration of insulin secretion through an anti-inflammatory action of oleic acid (65).

The effect of olive oil on circulating lipids has been extensively analyzed, in the context of the Mediterranean diet, taking into consideration their impact on cardiovascular diseases (54, 59) and the direct effect of olive oil is clearly demonstrated in performed meta-analyses (14, 22). In addition, George et al. (21) reported a moderate reduction of CVD risk by high-polyphenol olive oil, including effects on different oxidative parameters, total, HDL- and oxLDL-cholesterol. However, the authors reported the risk of biases, while the effect of the Mediterranean diet was not accessed. In the PREDIMED (13) and in a recent meta-analysis (13), the effect of HPOO as a protective agent in stroke but not in CVD was reported. Here, we show that the main effect of HPOO is the increase of circulating HDL, while other effects on cardio-metabolic parameters should be attributed to the Mediterranean diet *per se*. Additionally, the effect of olive oil on triglycerides might be mainly attributed to the lipid fraction than to microconstituents, as it is the same in high and low polyphenol content. These observed effects may be related to the reported impact of oleic acid and its metabolites, as well as of olive oil polyphenols on different enzymes, signaling molecules and a direct effect on the transcription of different proteins, including lipid-related, -transporting, or -metabolizing enzymes (31, 66–73).

Analyzing further the retained studies, we report (in a narrative review rather than through a meta-analysis, in view of the small number of studies) the effect of olive oil, high or not in antioxidant polyphenols, on oxidation and inflammation parameters. This effect of olive oil polyphenols has retained an increased attention, in view of the deleterious effect of oxidized lipids and nucleic acid damage, related to chronic diseases, including cardiovascular diseases and cancer (31, 67, 69, 71, 72, 74, 75). Analysis of our dataset confirmed the effect of olive oil polyphenols in protecting LDL and nucleic acid oxidation, in accordance to previous meta-analysis (76). However, an unexpected result reported here is that a much lower than the previously reported concentration of olive oil polyphenols is required to induce this protection (~60 mg/L of olive oil). This finding is in contradiction to the current belief that markers of oxidation (such as oxLDL and nucleic acid oxidation) are inversely related to the polyphenol content of olive oil, while the plasma antioxidant activity is directly related to it. The results of our analysis, if verified in prospective studies, could, therefore, provide a paradigm shift to currently established beliefs and might have a direct impact on the olive oil industry and human nutrition. It is further to note that the effect of olive oil on inflammatory markers is mainly evident in patients with an established metabolic syndrome (27), or in patients with cardiovascular disease (26), providing evidence of a possible protective/therapeutic use of olive oil in such conditions.

The novel element of the present meta-analysis consists of the estimation of LPOO and HPOO effects on cardio-metabolic parameters through a network analysis, estimating both their direct and indirect effects. Our data suggest that the major effect on these parameters is mediated through an adherence to the Mediterranean diet, while the only notable effect of olive oil polyphenols is the increase of HDL-cholesterol and the amelioration of the antioxidant and inflammatory status of the subjects. This effect is more pronounced in subjects with an established metabolic syndrome or other chronic conditions/diseases, evidencing its beneficial health effects. In addition, we report that much lower antioxidant polyphenols may be sufficient for the beneficial effects of olive oil, while we show that the lipid fraction of olive oil may be responsible for some of its beneficial actions. These conclusions, if verified in further prospective trials, may be of value in re-establishing the role of olive oil in human nutrition.

## Author Contributions

MK and EC conceived and designed the analysis and wrote the paper. ET, NP, and EC performed the analysis. All authors read and approved the final manuscript.

### Conflict of Interest Statement

The authors declare that the research was conducted in the absence of any commercial or financial relationships that could be construed as a potential conflict of interest.

## References

[1] Despres JP Lemieux I. Abdominal obesity and metabolic syndrome. Nature (2006) 444:881–7. 10.1038/nature05488 17167477

[2] Despres JP Lemieux I Bergeron J Pibarot P Mathieu P Larose E. Abdominal obesity and the metabolic syndrome: contribution to global cardiometabolic risk. Arterioscler Thromb Vasc Biol. (2008) 28:1039–49. 10.1161/ATVBAHA.107.159228 18356555

[3] Morange PE Alessi MC. Thrombosis in central obesity and metabolic syndrome: mechanisms and epidemiology. Thromb Haemost. (2013) 110:669–80. 10.1160/TH13-01-0075 23765199

[4] Keys A Mienotti A Karvonen MJ Aravanis C Blackburn H Buzina R. The diet and 15-year death rate in the seven countries study. Am J Epidemiol. (1986) 124:903–15. 10.1093/oxfordjournals.aje.a114480 3776973

[5] Pérez-Jiménez J Saura-Calixto F. Literature data may underestimate the actual antioxidant capacity of cereals. J Agric Food Chem. (2005) 53:5036–40. 10.1021/jf050049u 15941353

[6] Estruch R Martínez-González MA Corella D Salas-Salvad ó J Ruiz-Gutiérrez V Covas MI. (2006). Effects of a Mediterranean-style diet on cardiovascular risk factors: a randomized trial. Ann. Intern. Med. (2006) 145:1–11. 10.7326/0003-4819-145-1-200607040-00004 16818923

[7] Covas M-I. Olive oil and the cardiovascular system. Pharmacol Res. (2007) 55:175–86. 10.1016/j.phrs.2007.01.010 17321749

[8] Salas-Salvadó J Fernández-Ballart J Ros E Martínez-González MA Fitó M Estruch R. Effect of a Mediterranean diet supplemented with nuts on metabolic syndrome status: one-year results of the PREDIMED randomized trial. Arch. Intern. Med. (2008) 168:2449–58. 10.1001/archinte.168.22.2449 19064829

[9] Covas M-I Nyyssönen K Poulsen HE Kaikkonen J Zunft H-JF Kiesewetter H. The effect of polyphenols in olive oil on heart disease risk factors: a randomized trial. Ann Intern Med. (2006) 145:333–41. 10.7326/0003-4819-145-5-200609050-00006 16954359

[10] Panagiotakos DB Pitsavos C Polychronopoulos E Chrysohoou C Zampelas A Trichopoulou A. Can a Mediterranean diet moderate the development and clinical progression of coronary heart disease? A systematic review. Med Sci Monit. (2004) 10:RA193–8. 15278010

[11] Alonso A Ruiz-Gutierrez V Martinez-Gonzalez MA. Monounsaturated fatty acids, olive oil and blood pressure: epidemiological, clinical and experimental evidence. Public Health Nutr. (2006) 9:251–7. 10.1079/PHN2005836 16571180

[12] Berraaouan A Abid S Bnouham M. Antidiabetic oils. Curr Diabetes Rev. (2013) 9:499–505. 10.2174/15733998113096660081 24111621

[13] Martinez-Gonzalez MA Dominguez LJ Delgado-Rodriguez M. Olive oil consumption and risk of CHD and/or stroke: a meta-analysis of case-control, cohort and intervention studies. Br J Nutr. (2014) 112:248–59. 10.1017/S0007114514000713 24775425

[14] Schwingshackl L Hoffmann G. Monounsaturated fatty acids, olive oil and health status: a systematic review and meta-analysis of cohort studies. Lipids Health Dis. (2014) 13:154. 10.1186/1476-511X-13-154 25274026 PMC4198773

[15] Stradling C Hamid M Taheri S Thomas GN. A review of dietary influences on cardiovascular health: part 2: dietary patterns. Cardiovasc Hematol Disord Drug Targets (2014) 14:50–63. 10.2174/1871529X14666140701095426 24993125

[16] Vasto S Barera A Rizzo C Di Carlo M Caruso C Panotopoulos G. Mediterranean diet and longevity: an example of nutraceuticals? Curr Vasc Pharmacol. (2014) 12:735–8. 10.2174/1570161111666131219111818 24350926

[17] Martinez-Gonzalez MA Salas-Salvado J Estruch R Corella D Fito M Ros E. Benefits of the mediterranean diet: insights from the PREDIMED study. Prog Cardiovasc Dis. (2015) 58:50–60. 10.1016/j.pcad.2015.04.003 25940230

[18] Griffiths K Aggarwal BB Singh RB Buttar HS Wilson D De Meester F. Food antioxidants and their anti-inflammatory properties: a potential role in cardiovascular diseases and cancer prevention. Diseases (2016) 4:28. 10.3390/diseases4030028 28933408 PMC5456284

[19] Ahmad Farooqi A Fayyaz S Silva AS Sureda A Nabavi SF Mocan A. Oleuropein and cancer chemoprevention: the link is hot. Molecules (2017) 22:E705. 10.3390/molecules22050705 28468276 PMC6154543

[20] Schwingshackl L Lampousi AM Portillo MP Romaguera D Hoffmann G Boeing H. Olive oil in the prevention and management of type 2 diabetes mellitus: a systematic review and meta-analysis of cohort studies and intervention trials. Nutr Diabetes (2017) 7:e262. 10.1038/nutd.2017.12 28394365 PMC5436092

[21] George ES Marshall S Mayr HL Trakman GL Tatucu-Babet OA Lassemillante AM. The effect of high-polyphenol extra virgin olive oil on cardiovascular risk factors: a systematic review and meta-analysis. Crit Rev Food Sci Nutr. (2018). 10.1080/10408398.2018.1470491. [Epub ahead of print]. 29708409

[22] Ghobadi S Hassanzadeh-Rostami Z Mohammadian F Nikfetrat A Ghasemifard N Raeisi Dehkordi H. Comparison of blood lipid-lowering effects of olive oil and other plant oils: a systematic review and meta-analysis of 27 randomized placebo-controlled clinical trials. Crit Rev Food Sci Nutr. (2018). 10.1080/10408398.2018.1438349. [Epub ahead of print]. 29420053

[23] Zern TL Fernandez ML. Cardioprotective effects of dietary polyphenols. J Nutr. (2005) 135:2291–4. 10.1093/jn/135.10.2291 16177184

[24] Konstantinidou V Covas M-I MuñÎoz-Aguayo D Khymenets O De La Torre R Saez G. *In vivo* nutrigenomic effects of virgin olive oil polyphenols within the frame of the Mediterranean diet: a randomized controlled trial. FASEB J. (2010) 24:2546–57. 10.1096/fj.09-148452 20179144

[25] Cicero AF Nascetti S López-Sabater MC Elosua R Salonen JT NyyssőNen K. (2008). Changes in LDL fatty acid composition as a response to olive oil treatment are inversely related to lipid oxidative damage: the EUROLIVE study. J Am Coll Nutr. (2008) 27:314–320. 10.1080/07315724.2008.10719705 18689564

[26] Fitó M Cladellas M De La Torre R Marti J Munoz D Schröder H. Anti-inflammatory effect of virgin olive oil in stable coronary disease patients: a randomized, crossover, controlled trial. Eur J Clin Nutr. (2008) 62:570. 10.1038/sj.ejcn.1602724 17375118

[27] Esposito K Marfella R Ciotola M Di Palo C Giugliano F Giugliano G. Effect of a Mediterranean-style diet on endothelial dysfunction and markers of vascular inflammation in the metabolic syndrome: a randomized trial. JAMA (2004) 292:1440–6. 10.1001/jama.292.12.1440 15383514

[28] Chrysohoou C Panagiotakos DB Pitsavos C Das UN Stefanadis C. Adherence to the Mediterranean diet attenuates inflammation and coagulation process in healthy adults: the ATTICA Study. J Am Coll Cardiol. (2004) 44:152–8. 10.1016/j.jacc.2004.03.039 15234425

[29] Marrugat J Covas MI Fitó M Schröder H Miró-Casas E Gimeno E. Effects of differing phenolic content in dietary olive oils on lipids and LDL oxidation. Eur J Nutr. (2004) 43:140–7. 10.1007/s00394-004-0452-8 15168036

[30] Aviram M Eias K. Dietary olive oil reduces low-density lipoprotein uptake by macrophages and decreases the susceptibility of the lipoprotein to undergo lipid peroxidation. Ann Nutr Metab. (1993) 37:75–84. 10.1159/000177753 8517637

[31] Visioli F Caruso D Grande S Bosisio R Villa M Galli G. Virgin Olive Oil Study (VOLOS): vasoprotective potential of extra virgin olive oil in mildly dyslipidemic patients. Eur J Nutr. (2005) 44:121–7. 10.1007/s00394-004-0504-0 15309433

[32] Vissers MN Zock PL Wiseman SA Meyboom S Katan MB. Effect of phenol-rich extra virgin olive oil on markers of oxidation in healthy volunteers. Eur J Clin Nutr. (2001) 55:334. 10.1038/sj.ejcn.1601161 11378806

[33] Ramirez-Tortosa MC Urbano G López-Jurado M Nestares T Gomez MC Mir A. Extra-virgin olive oil increases the resistance of LDL to oxidation more than refined olive oil in free-living men with peripheral vascular disease. J Nutr. (1999) 129:2177–83. 10.1093/jn/129.12.2177 10573546

[34] Masella R Giovannini C Varì R Di Benedetto R Coni E Volpe R. Effects of dietary virgin olive oil phenols on low density lipoprotein oxidation in hyperlipidemic patients. Lipids (2001) 36:1195–202. 10.1007/s11745-001-0832-3 11795851

[35] Visioli F Caruso D Galli C Viappiani S Galli G Sala A. Olive oils rich in natural catecholic phenols decrease isoprostane excretion in humans. Biochem Biophys Res Commun. (2000) 278:797–9. 10.1006/bbrc.2000.3879 11095986

[36] Bogani P Galli C Villa M Visioli F. Postprandial anti-inflammatory and antioxidant effects of extra virgin olive oil. Atherosclerosis (2007) 190:181–6. 10.1016/j.atherosclerosis.2006.01.011 16488419

[37] Weinbrenner T Fito M De La Torre R Saez GT Rijken P Tormos C. Olive oils high in phenolic compounds modulate oxidative/antioxidative status in men. J Nutr. (2004) 134:2314–21. 10.1093/jn/134.9.2314 15333722

[38] Machowetz A Poulsen HE Gruendel S Weimann A Fitó M Marrugat J. Effect of olive oils on biomarkers of oxidative DNA stress in Northern and Southern Europeans. FASEB J. (2007) 21:45–52. 10.1096/fj.06-6328com 17110467

[39] Moschandreas J Vissers M Wiseman S Van Putte K Kafatos A. Extra virgin olive oil phenols and markers of oxidation in Greek smokers: a randomized cross-over study. Eur J Clin Nutr. (2002) 56:1024. 10.1038/sj.ejcn.1601444 12373624

[40] Reaven PD Grasse BJ Tribble DL. Effects of linoleate-enriched and oleate-enriched diets in combination with alpha-tocopherol on the susceptibility of LDL and LDL subfractions to oxidative modification in humans. Arterioscler Thromb Vasc Biol. (1994) 14:557–66. 10.1161/01.ATV.14.4.557 8148354

[41] Ruano J Lopez-Miranda J Fuentes F Moreno JA Bellido C Perez-Martinez P. Phenolic content of virgin olive oil improves ischemic reactive hyperemia in hypercholesterolemic patients. J Am Coll Cardiol. (2005) 46:1864–8. 10.1016/j.jacc.2005.06.078 16286173

[42] Salvini S Sera F Caruso D Giovannelli L Visioli F Saieva C. Daily consumption of a high-phenol extra-virgin olive oil reduces oxidative DNA damage in postmenopausal women. Br J Nutr. (2006) 95:742–51. 10.1079/BJN20051674 16571154

[43] Wagner K-H Tomasch R Elmadfa I. Impact of diets containing corn oil or olive/sunflower oil mixture on the human plasma and lipoprotein lipid metabolism. Eur J Nutr. (2001) 40:161–7. 10.1007/s003940170004 11905957

[44] Tholstrup T Hjerpsted J Raff M. Palm olein increases plasma cholesterol moderately compared with olive oil in healthy individuals. Am J Clin Nutr. (2011) 94:1426–32. 10.3945/ajcn.111.018846 22071711

[45] Hernáez Á Fernández-Castillejo S Farràs M Catalán Ú Subirana I Montes R (2014). Olive oil polyphenols enhance high-density lipoprotein function in humans: a randomized controlled trial. Arterioscler Thromb Vasc Biol. (2014) 114 303374 10.1161/ATVBAHA.114.303374 25060792

[46] Anderson-Vasquez HE Pérez-Martínez P Ortega Fernández P Wanden-Berghe C. Impact of the consumption of a rich diet in butter and it replacement for a rich diet in extra virgin olive oil on anthropometric, metabolic and lipid profile in postmenopausal women. Nutr Hosp. (2015) 31:2561–70. 10.3305/nh.2015.31.6.8732 26040366

[47] Venturini D Urbano MR Dichi I. Effects of extra virgin olive oil and fish oil on lipid profile and oxidative stress in patients with metabolic syndrome. Nutrition (2015) 31:834–40. 10.1016/j.nut.2014.12.016 25933490

[48] Farràs M Castañer O Martín-Peláez S Hernáez Á Schröder H Subirana I. Complementary phenol-enriched olive oil improves HDL characteristics in hypercholesterolemic subjects. A randomized, double-blind, crossover, controlled trial. The VOHF study. Mol Nutr Food Res. (2015) 59:1758–70. 10.1002/mnfr.201500030 26011257

[49] Hernáez Á Remaley AT Farràs M Fernández-Castillejo S Subirana I Schröder H Olive oil polyphenols decrease LDL concentrations and LDL atherogenicity in men in a randomized controlled trial−3. J Nutr. (2015) 145:1692–7. 10.3945/jn.115.211557 26136585 PMC4516770

[50] Kastorini CM Panagiotakos D Georgousopoulou E Laskaris A Skourlis N Zana A. Metabolic syndrome and 10-year cardiovascular disease incidence: the ATTICA study. Nutr Metab Cardiovasc Dis. (2016) 26:223–31. 10.1016/j.numecd.2015.12.010 26803591

[51] Pedret A Catalan U Fernandez-Castillejo S Farràs M Valls RM Rubió L. (2015). Impact of virgin olive oil and phenol-enriched virgin olive oils on the HDL proteome in hypercholesterolemic subjects: a double blind, randomized, controlled, cross-over clinical trial (VOHF study). PLoS ONE (2015) 10:e0129160. 10.1371/journal.pone.0129160 26061039 PMC4465699

[52] Bondia-Pons I Schröder H Covas MI Castellote AI Kaikkonen J Poulsen HE. Moderate consumption of olive oil by healthy European men reduces systolic blood pressure in non-Mediterranean participants. J Nutr. (2007) 137:84–7. 10.1093/jn/137.1.84 17182805

[53] Salanti G Del Giovane C Chaimani A Caldwell DM Higgins JP. Evaluating the quality of evidence from a network meta-analysis. PLoS ONE (2014) 9:e99682. 10.1371/journal.pone.0099682 24992266 PMC4084629

[54] Nadtochiy SM Redman EK. Mediterranean diet and cardioprotection: the role of nitrite, polyunsaturated fatty acids, and polyphenols. Nutrition (2011) 27:733–44. 10.1016/j.nut.2010.12.006 21454053 PMC3117983

[55] Diez-Espino J Buil-Cosiales P Serrano-Martinez M Toledo E Salas-Salvado J Martinez-Gonzalez MA. Adherence to the Mediterranean diet in patients with type 2 diabetes mellitus and HbA1c level. Ann Nutr Metab. (2011) 58:74–8. 10.1159/000324718 21430378

[56] Marin C Ramirez R Delgado-Lista J Yubero-Serrano EM Perez-Martinez P Carracedo J. Mediterranean diet reduces endothelial damage and improves the regenerative capacity of endothelium. Am J Clin Nutr. (2011) 93:267–74. 10.3945/ajcn.110.006866 21123460

[57] Perez-Martinez P Garcia-Rios A Delgado-Lista J Perez-Jimenez F Lopez-Miranda J. Mediterranean diet rich in olive oil and obesity, metabolic syndrome and diabetes mellitus. Curr Pharm Des. (2011) 17:769–77. 10.2174/138161211795428948 21443484

[58] Salas-Salvado J Guasch-Ferre M Lee CH Estruch R Clish CB Ros E (2016). Protective effects of the mediterranean diet on type 2 diabetes and metabolic syndrome. J Nutr. 146:920S–7S. 10.3945/jn.115.218487 PMC4807638 26962178

[59] Damasceno NR Perez-Heras A Serra M Cofan M Sala-Vila A Salas-Salvado J. Crossover study of diets enriched with virgin olive oil, walnuts or almonds. Effects on lipids and other cardiovascular risk markers. Nutr Metab Cardiovasc Dis. (2011) 21 (Suppl. 1):S14–20. 10.1016/j.numecd.2010.12.006 21421296

[60] Kastorini CM Milionis HJ Esposito K Giugliano D Goudevenos JA Panagiotakos DB. The effect of Mediterranean diet on metabolic syndrome and its components: a meta-analysis of 50 studies and 534,906 individuals. J Am Coll Cardiol. (2011) 57:1299–313. 10.1016/j.jacc.2010.09.073 21392646

[61] Casas R Sacanella E Urpi-Sarda M Corella D Castaner O Lamuela-Raventos RM. Long-term immunomodulatory effects of a mediterranean diet in adults at high risk of cardiovascular disease in the PREvencion con DIeta MEDiterranea (PREDIMED) randomized controlled trial. J Nutr. (2016) 146:1684–93. 10.3945/jn.115.229476 27440261

[62] Efsa Panel on Dietetic Products Nutrition and Allergies (NDA) Scientific Opinion on the substantiation of health claims related to olive oil and maintenance of normal blood LDL-cholesterol concentrations (ID 1316:1332), maintenance of normal (fasting) blood concentrations of triglycerides (ID 1316:1332), maintenance of normal blood HDL cholesterol concentrations (ID 1316:1332) and maintenance of normal blood glucose concentrations (ID 4244) pursuant to Article 13(1) of Regulation (EC) No 1924/2006. EFSA J. (2011) 9:2044 10.2903/j.efsa.2011.2044 PMC13129925 42078784

[63] Peroulis N Androutsopoulos VP Notas G Koinaki S Giakoumaki E Spyros A. Significant metabolic improvement by a water extract of olives: animal and human evidence. Eur J Nutr. (2018). 10.1007/s00394-018-1807-x. [Epub ahead of print]. 30094646

[64] Lee H Im SW Jung CH Jang YJ Ha TY Ahn J. Tyrosol, an olive oil polyphenol, inhibits ER stress-induced apoptosis in pancreatic beta-cell through JNK signaling. Biochem Biophys Res Commun. (2016) 469:748–52. 10.1016/j.bbrc.2015.12.036 26692476

[65] Vassiliou EK Gonzalez A Garcia C Tadros JH Chakraborty G Toney JH. Oleic acid and peanut oil high in oleic acid reverse the inhibitory effect of insulin production of the inflammatory cytokine TNF-alpha both in vitro and *In vivo* systems. Lipids Health Dis. (2009) 8:25. 10.1186/1476-511X-8-25 19558671 PMC2706835

[66] Rahman MH Avella MA Botham KM. The fatty acid composition of chylomicrons influences the rate of their lipolysis *in vivo*. Nutr Metab Cardiovasc Dis. (2000) 10:121–5. 11006920

[67] Carluccio MA Massaro M Scoditti E De Caterina R. Vasculoprotective potential of olive oil components. Mol Nutr Food Res. (2007) 51:1225–34. 10.1002/mnfr.200600305 17912721

[68] Alemany R Navarro MA Vogler O Perona JS Osada J Ruiz-Gutierrez V. Olive oils modulate fatty acid content and signaling protein expression in apolipoprotein E knockout mice brain. Lipids (2010) 45:53–61. 10.1007/s11745-009-3370-y 19924462

[69] Llorente-Cortes V Estruch R Mena MP Ros E Gonzalez MA Fito M. Effect of Mediterranean diet on the expression of pro-atherogenic genes in a population at high cardiovascular risk. Atherosclerosis (2010) 208:442–50. 10.1016/j.atherosclerosis.2009.08.004 19712933

[70] Gabas-Rivera C Martinez-Beamonte R Rios JL Navarro MA Surra JC Arnal C. Dietary oleanolic acid mediates circadian clock gene expression in liver independently of diet and animal model but requires apolipoprotein A1. J Nutr Biochem. (2013) 24:2100–9. 10.1016/j.jnutbio.2013.07.010 24231102

[71] Lopez S Jaramillo S Varela LM Ortega A Bermudez B Abia R. p38 MAPK protects human monocytes from postprandial triglyceride-rich lipoprotein-induced toxicity. J Nutr. (2013) 143:620–6. 10.3945/jn.113.174656 23486980

[72] Loued S Berrougui H Componova P Ikhlef S Helal O Khalil A. Extra-virgin olive oil consumption reduces the age-related decrease in HDL and paraoxonase 1 anti-inflammatory activities. Br J Nutr. (2013) 110:1272–84. 10.1017/S0007114513000482 23510814

[73] Dunbar RL Nicholls SJ Maki KC Roth EM Orloff DG Curcio D. Effects of omega-3 carboxylic acids on lipoprotein particles and other cardiovascular risk markers in high-risk statin-treated patients with residual hypertriglyceridemia: a randomized, controlled, double-blind trial. Lipids Health Dis. (2015) 14:98. 10.1186/s12944-015-0100-8 26328624 PMC4557761

[74] Rolandsson O Hagg E Nilsson M Hallmans G Mincheva-Nilsson L Lernmark A. Prediction of diabetes with body mass index, oral glucose tolerance test and islet cell autoantibodies in a regional population. J Intern Med. (2001) 249:279–88. 10.1046/j.1365-2796.2001.00813.x 11298847

[75] Diniz YS Cicogna AC Padovani CR Santana LS Faine LA Novelli EL. Diets rich in saturated and polyunsaturated fatty acids: metabolic shifting and cardiac health. Nutrition (2004) 20:230–4. 10.1016/j.nut.2003.10.012 14962692

[76] Schwingshackl L Christoph M Hoffmann G. Effects of olive oil on markers of inflammation and endothelial function-a systematic review and meta-analysis. Nutrients (2015) 7:7651–75. 10.3390/nu7095356 26378571 PMC4586551

