# Supplementary material for: Network Meta-Analysis of Metabolic Effects of Olive-Oil in Humans Shows the Importance of Olive Oil Consumption With Moderate Polyphenol Levels as Part of the Mediterranean Diet
Source: Front Nutr. 2019 Feb 12;6:6. doi: 10.3389/fnut.2019.00006 (PMC6379345; doi:10.3389/fnut.2019.00006)
Supplement: Supplementary file 1 [file Data_Sheet_1.docx]

**Supplemental Material**

**Supplemental Table 1**

Direct, indirect and total effects of Mediterranean Diet, Low and high polyphenol olive oil (LPOO and HPOO respectively) on circulating glucose levels. Results are presented and standardized differences between comparison groups, together with their 95% confidence interval. Number in parentheses refer to the models ID used for the calculations. MD=Mediterranean Diet; LPOO and PL=low polyphenol olive oil, HPOO and HP=high polyphenol olive oil. Figure presents the different comparisons presented in the Table. Each intervention, including the comparator, is represented by a circle. Interventions that have been directly compared in the input studies are connected with a line. The width of that line is proportional to the number of studies that compared the two interventions. The size of the intervention circle is proportional to the number of arms that intervention occurs across all input studies. The same comparisons have also be made in Tables 2-6.

| **ID** | **Comparison** | **Intervention** | **Control** | **d** | **LCI 95%** | **HCI 95%** |
| --- | --- | --- | --- | --- | --- | --- |
|  | **Direct estimates** |  |  |  |  |  |
| 1 | Glu-MD-C | MedDiet | Control | -0.112 | -0.182 | -0.042 |
| 2 | Glu-LPOO-C | LPOO | Control | 0.044 | -0.185 | 0.273 |
| 3 | Glu-HPOO-C | HPOO | Control | 0.085 | -0.057 | 0.226 |
| 4 | Glu-HP-LP | HPOO | LPOO | 0.039 | -0.325 | 0.403 |
| 5 | Glu-MD-LP | LPOO | MedDiet | 0.000 | -0.438 | 0.438 |
| 6 | Glu-MD-HP | HPOO | MedDiet | 0.000 | -0.438 | 0.438 |
|  |  |  |  |  |  |  |
|  | **Indirect estimates (source IDs)** |  |  |  |  |  |
| 7 | Indirect HPOO vs Control (4, 2) | HPOO | Control | 0.083 | -0.347 | 0.513 |
| 8 | Indirect LPOO vs Control (5, 1) | LPOO | Control | -0.112 | -0.556 | 0.332 |
| 9 | Indirect HPOO vs Control (6, 1) | HPOO | Control | -0.112 | -0.556 | 0.332 |
| 10 | Indirect MedDiet vs Control (5, 2) | MedDiet | Control | 0.044 | -0.450 | 0.538 |
| 11 | Indirect LPOO vs Control (4, 3) | LPOO | Control | 0.046 | -0.345 | 0.436 |
| 12 | Indirect MedDiet vs Control (6, 3) | MedDiet | Control | 0.085 | -0.376 | 0.545 |
|  |  |  |  |  |  |  |
|  | **Result estimates (source IDs)** |  |  |  |  |  |
|  | MedDiet (1, 10, 12) | MedDiet | Control | -0.105 | -0.174 | -0.036 |
|  | LPOO (2, 8, 11) | LPOO | Control | 0.019 | -0.162 | 0.199 |
|  | HPOO (3, 7, 9) | HPOO | Control | 0.068 | -0.061 | 0.197 |

**Supplemental Table 2**

Direct, indirect and total effects of Mediterranean Diet, Low and high polyphenol olive oil (LPOO and HPOO respectively) on circulating triglyceride levels. Results are presented and standardized differences between comparison groups, together with their 95% confidence interval. Number in parentheses refer to the model ID used for the calculations.

| **ID** | **Comparison** | **Intervention** | **Control** | **d** | **LCI 95%** | **HCI 95%** |
| --- | --- | --- | --- | --- | --- | --- |
|  | **Direct estimates** |  |  |  |  |  |
| 1 | TG-MD-C | MedDiet | Control | -0.131 | -0.201 | -0.061 |
| 2 | TG-LPOO-C | LPOO | Control | -0.009 | -0.138 | 0.121 |
| 3 | TG-HPOO-C | HPOO | Control | -0.033 | -0.142 | 0.076 |
| 4 | TG-HP-LP | HPOO | LPOO | -0.018 | -0.169 | 0.134 |
| 5 | TG-MD-LP | LPOO | MedDiet | 0.032 | -0.407 | 0.470 |
| 6 | TG-MD-HP | HPOO | MedDiet | -0.047 | -0.486 | 0.391 |
|  |  |  |  |  |  |  |
|  | **Indirect estimates (source IDs)** |  |  |  |  |  |
| 7 | Indirect HPOO vs Control (4, 2) | HPOO | Control | -0.027 | -0.226 | 0.173 |
| 8 | Indirect LPOO vs Control (5, 1) | LPOO | Control | -0.100 | -0.544 | 0.344 |
| 9 | Indirect HPOO vs Control (6, 1) | HPOO | Control | -0.179 | -0.623 | 0.265 |
| 10 | Indirect MedDiet vs Control (5, 2) | MedDiet | Control | -0.040 | -0.497 | 0.417 |
| 11 | Indirect LPOO vs Control (4, 3) | LPOO | Control | -0.015 | -0.202 | 0.172 |
| 12 | Indirect MedDiet vs Control (6, 3) | MedDiet | Control | 0.014 | -0.437 | 0.466 |
|  |  |  |  |  |  |  |
|  | **Result estimates (source IDs)** |  |  |  |  |  |
|  | MedDiet (1, 10, 12) | MedDiet | Control | -0.126 | -0.194 | -0.057 |
|  | LPOO (2, 8, 11) | LPOO | Control | -0.016 | -0.119 | 0.088 |
|  | HPOO (3, 7, 9) | HPOO | Control | -0.038 | -0.132 | 0.056 |

**Supplemental Table 3**

Direct, indirect and total effects of Mediterranean Diet, Low and high polyphenol olive oil (LPOO and HPOO respectively) on circulating total cholesterol (TC) levels. Results are presented and standardized differences between comparison groups, together with their 95% confidence interval. Number in parentheses refer to the model ID used for the calculations.

| **ID** | **Comparison** | **Intervention** | **Control** | **d** | **LCI 95%** | **HCI 95%** |
| --- | --- | --- | --- | --- | --- | --- |
|  | **Direct estimates** |  |  |  |  |  |
| 1 | TC-MD-C | MedDiet | Control | -0.195 | -0.265 | -0.124 |
| 2 | TC-LPOO-C | LPOO | Control | -0.136 | -0.255 | -0.017 |
| 3 | TC-HPOO-C | HPOO | Control | -0.051 | -0.152 | 0.051 |
| 4 | TC-HP-LP | HPOO | LPOO | 0.027 | -0.125 | 0.178 |
| 5 | TC-MD-LP | LPOO | MedDiet | -0.043 | -0.481 | 0.395 |
| 6 | TC-MD-HP | HPOO | MedDiet | 0.066 | -0.373 | 0.504 |
|  |  |  |  |  |  |  |
|  | **Indirect estimates (source IDs)** |  |  |  |  |  |
| 7 | Indirect HPOO vs Control (4, 2) | HPOO | Control | -0.109 | -0.302 | 0.083 |
| 8 | Indirect LPOO vs Control (5, 1) | LPOO | Control | -0.238 | -0.681 | 0.206 |
| 9 | Indirect HPOO vs Control (6, 1) | HPOO | Control | -0.129 | -0.573 | 0.315 |
| 10 | Indirect MedDiet vs Control (5, 2) | MedDiet | Control | -0.093 | -0.547 | 0.361 |
| 11 | Indirect LPOO vs Control (4, 3) | LPOO | Control | -0.077 | -0.259 | 0.105 |
| 12 | Indirect MedDiet vs Control (6, 3) | MedDiet | Control | -0.116 | -0.566 | 0.334 |
|  |  |  |  |  |  |  |
|  | **Result estimates (source IDs)** |  |  |  |  |  |
|  | MedDiet (1, 10, 12) | MedDiet | Control | -0.191 | -0.259 | -0.122 |
|  | LPOO (2, 8, 11) | LPOO | Control | -0.124 | -0.221 | -0.027 |
|  | HPOO (3, 7, 9) | HPOO | Control | -0.066 | -0.154 | 0.022 |

**Supplemental Table 4**

Direct, indirect and total effects of Mediterranean Diet, Low and high polyphenol olive oil (LPOO and HPOO respectively) on circulating HDL-cholesterol levels. Results are presented and standardized differences between comparison groups, together with their 95% confidence interval. Number in parentheses refer to the model ID used for the calculations.

| **ID** | **Comparison** | **Intervention** | **Control** | **d** | **LCI 95%** | **HCI 95%** |
| --- | --- | --- | --- | --- | --- | --- |
|  | **Direct estimates** |  |  |  |  |  |
| 1 | HDL-MD-C | MedDiet | Control | 0.113 | 0.062 | 0.163 |
| 2 | HDL-LPOO-C | LPOO | Control | 0.130 | 0.011 | 0.248 |
| 3 | HDL-HPOO-C | HPOO | Control | 0.157 | 0.056 | 0.258 |
| 4 | HDL-HP-LP | HPOO | LPOO | 0.086 | -0.065 | 0.238 |
| 5 | HDL-MD-LP | LPOO | MedDiet | 0.000 | -0.438 | 0.438 |
| 6 | HDL-MD-HP | HPOO | MedDiet | 0.000 | -0.438 | 0.438 |
|  |  |  |  |  |  |  |
|  | **Indirect estimates (source IDs)** |  |  |  |  |  |
| 7 | Indirect HPOO vs Control (4, 2) | HPOO | Control | 0.216 | 0.024 | 0.409 |
| 8 | Indirect LPOO vs Control (5, 1) | LPOO | Control | 0.113 | -0.329 | 0.554 |
| 9 | Indirect HPOO vs Control (6, 1) | HPOO | Control | 0.113 | -0.329 | 0.554 |
| 10 | Indirect MedDiet vs Control (5, 2) | MedDiet | Control | 0.130 | -0.324 | 0.584 |
| 11 | Indirect LPOO vs Control (4, 3) | LPOO | Control | 0.070 | -0.112 | 0.252 |
| 12 | Indirect MedDiet vs Control (6, 3) | MedDiet | Control | 0.157 | -0.293 | 0.606 |
|  |  |  |  |  |  |  |
|  | **Result estimates (source IDs)** |  |  |  |  |  |
|  | MedDiet (1, 10, 12) | MedDiet | Control | 0.113 | 0.064 | 0.163 |
|  | LPOO (2, 8, 11) | LPOO | Control | 0.112 | 0.015 | 0.209 |
|  | HPOO (3, 7, 9) | HPOO | Control | 0.167 | 0.080 | 0.255 |

**Supplemental Table 5**

Direct, indirect and total effects of Mediterranean Diet, Low and high polyphenol olive oil (LPOO and HPOO respectively) on circulating LDL-cholesterol levels. Results are presented and standardized differences between comparison groups, together with their 95% confidence interval. Number in parentheses refer to the model ID used for the calculations.

| **ID** | **Comparison** | **Intervention** | **Control** | **d** | **LCI 95%** | **HCI 95%** |
| --- | --- | --- | --- | --- | --- | --- |
|  | **Direct estimates** |  |  |  |  |  |
| 1 | LDL-MD-C | MedDiet | Control | -0.190 | -0.240 | -0.140 |
| 2 | LDL-LPOO-C | LPOO | Control | -0.161 | -0.280 | -0.042 |
| 3 | LDL-HPOO-C | HPOO | Control | -0.074 | -0.182 | 0.033 |
| 4 | LDL-HP-LP | HPOO | LPOO | -0.015 | -0.166 | 0.137 |
| 5 | LDL-MD-LP | LPOO | MedDiet | -0.050 | -0.488 | 0.388 |
| 6 | LDL-MD-HP | HPOO | MedDiet | 0.074 | -0.364 | 0.513 |
|  |  |  |  |  |  |  |
|  | **Indirect estimates (source IDs)** |  |  |  |  |  |
| 7 | Indirect HPOO vs Control (4, 2) | HPOO | Control | -0.176 | -0.369 | 0.017 |
| 8 | Indirect LPOO vs Control (5, 1) | LPOO | Control | -0.240 | -0.682 | 0.201 |
| 9 | Indirect HPOO vs Control (6, 1) | HPOO | Control | -0.116 | -0.557 | 0.325 |
| 10 | Indirect MedDiet vs Control (5, 2) | MedDiet | Control | -0.111 | -0.565 | 0.343 |
| 11 | Indirect LPOO vs Control (4, 3) | LPOO | Control | -0.059 | -0.245 | 0.127 |
| 12 | Indirect MedDiet vs Control (6, 3) | MedDiet | Control | -0.148 | -0.600 | 0.303 |
|  |  |  |  |  |  |  |
|  | **Result estimates (source IDs)** |  |  |  |  |  |
|  | MedDiet (1, 10, 12) | MedDiet | Control | -0.189 | -0.238 | -0.140 |
|  | LPOO (2, 8, 11) | LPOO | Control | -0.137 | -0.234 | -0.039 |
|  | HPOO (3, 7, 9) | HPOO | Control | -0.099 | -0.191 | -0.007 |

**Supplemental Table 6**

Direct, indirect and total effects of Mediterranean Diet, Low and high polyphenol olive oil (LPOO and HPOO respectively) on circulating oxidized LDL (oxL) levels. Results are presented and standardized differences between comparison groups, together with their 95% confidence interval. Number in parentheses refer to the model ID used for the calculations.

| **ID** | **Comparison** | **Active** | **Control** | **d** | **LCI 95%** | **HCI 95%** |
| --- | --- | --- | --- | --- | --- | --- |
|  | **Direct estimates** |  |  |  |  |  |
| 1 | oxL-MD-C | MedDiet | Control | -0.228 | -0.668 | 0.211 |
| 2 | oxL-LPOO-C | LPOO | Control | -0.001 | -0.173 | 0.171 |
| 3 | oxL-HPOO-C | HPOO | Control | -0.094 | -0.217 | 0.030 |
| 4 | oxL-HP-LP | HPOO | LPOO | 0.105 | -0.067 | 0.277 |
| 5 | oxL-MD-LP | LPOO | MedDiet | 0.044 | -0.394 | 0.482 |
| 6 | oxL-MD-HP | HPOO | MedDiet | -0.043 | -0.481 | 0.395 |
|  |  |  |  |  |  |  |
|  | **Indirect estimates (source IDs)** |  |  |  |  |  |
| 7 | Indirect HPOO vs Control (4, 2) | HPOO | Control | 0.104 | -0.140 | 0.347 |
| 8 | Indirect LPOO vs Control (5, 1) | LPOO | Control | -0.184 | -0.805 | 0.437 |
| 9 | Indirect HPOO vs Control (6, 1) | HPOO | Control | -0.271 | -0.892 | 0.350 |
| 10 | Indirect MedDiet vs Control (5, 2) | MedDiet | Control | -0.045 | -0.516 | 0.425 |
| 11 | Indirect LPOO vs Control (4, 3) | LPOO | Control | -0.199 | -0.410 | 0.013 |
| 12 | Indirect MedDiet vs Control (6, 3) | MedDiet | Control | -0.051 | -0.506 | 0.405 |
|  |  |  |  |  |  |  |
|  | **Result estimates (source IDs)** |  |  |  |  |  |
|  | MedDiet (1, 10, 12) | MedDiet | Control | -0.112 | -0.375 | 0.150 |
|  | LPOO (2, 8, 11) | LPOO | Control | -0.084 | -0.222 | 0.053 |
|  | HPOO (3, 7, 9) | HPOO | Control | -0.060 | -0.206 | 0.086 |

**Supplemental Figure 1**

Meta-analysis of circulating glucose levels in the 30 studies presented in Table 1. Data are presented as the Cohen standardized difference d (the difference between the means divided by the standard deviation for the data), together with 95% confidence interval (95% CI). Med.Diet=Mediterranean diet; HPOO and LPOO=high- and low-polyphenol olive oil respectively.

**Supplemental Figure 2**

Meta-analysis of circulating triglyceride levels in the 30 studies presented in Table 1. Data are presented as the Cohen standardized difference d (the difference between the means divided by the standard deviation for the data), together with 95% confidence interval (95% CI). Med.Diet=Mediterranean diet; HPOO and LPOO=high- and low-polyphenol olive oil respectively.

**Supplemental Figure 3**

Meta-analysis of circulating total cholesterol levels in the 30 studies presented in Table 1. Data are presented as the Cohen standardized difference d (the difference between the means divided by the standard deviation for the data), together with 95% confidence interval (95% CI). Med.Diet=Mediterranean diet; HPOO and LPOO=high- and low-polyphenol olive oil respectively.

**Supplemental Figure 4**

Meta-analysis of circulating HDL-cholesterol levels in the 30 studies presented in Table 1. Data are presented as the Cohen standardized difference d (the difference between the means divided by the standard deviation for the data), together with 95% confidence interval (95% CI). Med.Diet=Mediterranean diet; HPOO and LPOO=high- and low-polyphenol olive oil respectively.

**Supplemental Figure 5**

Meta-analysis of circulating LDL-cholesterol levels in the 30 studies presented in Table 1. Data are presented as the Cohen standardized difference d (the difference between the means divided by the standard deviation for the data), together with 95% confidence interval (95% CI). Med.Diet=Mediterranean diet; HPOO and LPOO=high- and low-polyphenol olive oil respectively.

**Supplemental Figure 6**

Meta-analysis of circulating oxidized LDL-cholesterol levels in the 30 studies presented in Table 1. Data are presented as the Cohen standardized difference d (the difference between the means divided by the standard deviation for the data), together with 95% confidence interval (95% CI). Med.Diet=Mediterranean diet; HPOO and LPOO=high- and low-polyphenol olive oil respectively.
